# A data-calibrated model of dynamic flow diversions to AI-assisted teledermatology

**DOI:** 10.1016/j.jdin.2026.09.005

**Published:** 2026-09-28

**Authors:** Abraham Seidmann, Fanying Chen, Opher Baron, Avi Porath

**Affiliations:** a Boston University, Questrom School of Business, Boston, Massachusetts; b Rotman School of Management, University of Toronto, and SiMLQ, Toronto, Ontario, Canada; c Maccabi HMO, Israel & Ben Gurion University, Tel Aviv, Israel

**Keywords:** general dermatology, health IT, medical dermatology, teledermatology, workflow operations

**Figure:**
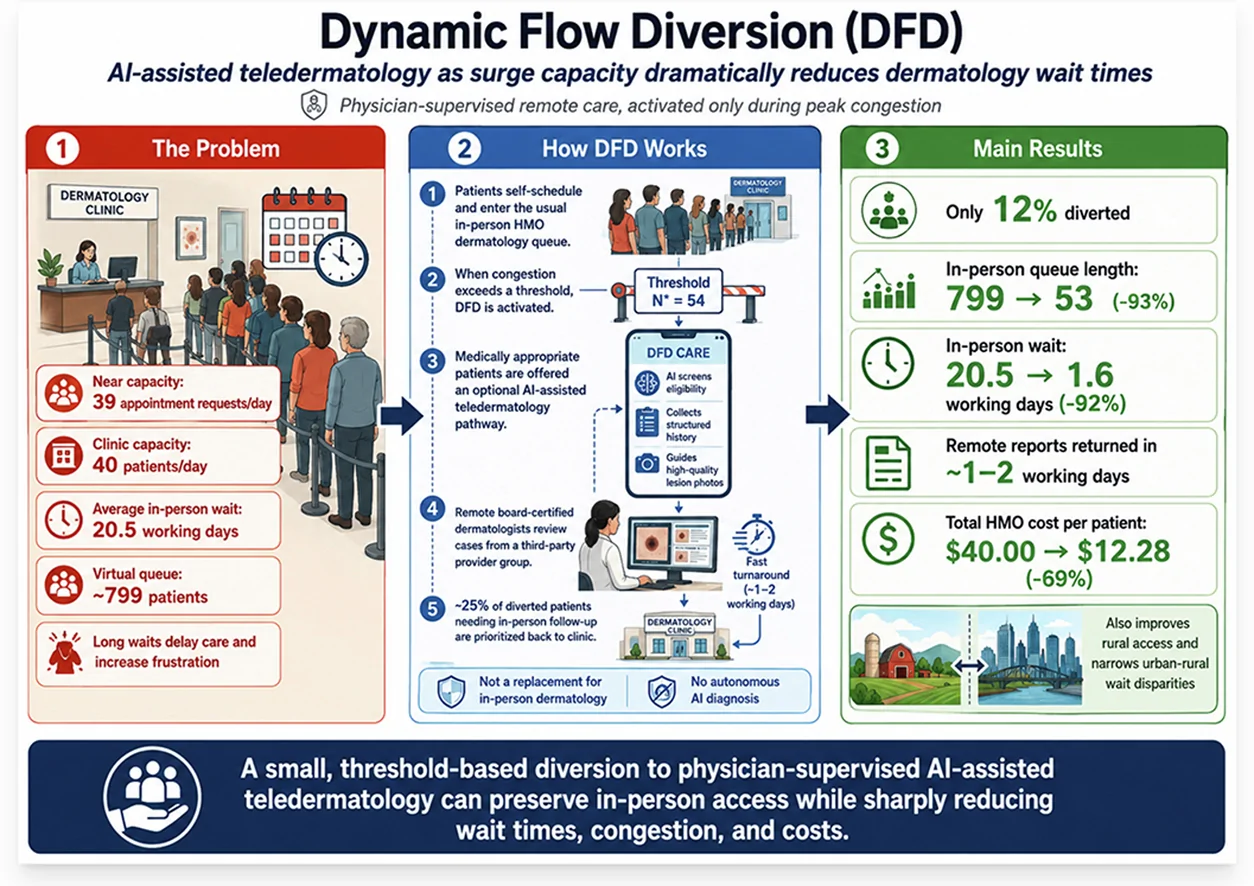

*To the Editor:* Long waits for dermatology care can significantly delay time-sensitive diagnoses, increase patient frustration, worsen nonattendance, and lead to unnecessary visits to other clinicians. The problem is especially severe at clinics operating near full capacity; in such settings, even small increases in demand will produce large increases in waiting times.^1^

We propose a practical clinical workflow that uses artificial intelligence (AI)-assisted teledermatology,^2^ only as a surge capacity. The goal is not to replace in-person dermatology or allow AI to diagnose independently. Instead, the system adds a supervised remote pathway that is activated only when the in-person dermatology queue exceeds a predetermined economic threshold, see Table I.

**Table I:** Practical design principles for AI-assisted teledermatology surge capacity

Operational problem	Proposed design response
Long dermatology waits	Activate remote capacity only when the queue length at a particular clinic exceeds a well-defined economic threshold
Limited dermatologist supply	Use an external board-certified dermatology group as surge capacity, to review images remotely, and pay them on a per-case basis
Concern about AI safety	Use AI for intake, image quality, and routing, not for any autonomous diagnosis
Risk of fragmented care	Prioritize patients needing follow-up back into the in-person HMO clinic

*HMO*, Health Maintenance Organization.

In the dynamic flow diversion (DFD) model depicted in Fig 1, patients who self-schedule dermatology appointments online remain in the usual virtual queue to be seen by salaried Health Maintenance Organization (HMO) dermatologists. However, when congestion exceeds a preset threshold, medically appropriate patients are automatically offered an optional AI-assisted teledermatology visit. The AI supports case info intake by patients, and image quality rather than diagnosis.^3^ The external third-party dermatologists review and escalate all appropriate cases. This external group is paid per use and contracted for rapid turnaround times. This fee-for-service contract also avoids fixed costs when demand subsides. The DFD system prioritizes patients requiring in-person follow-up for return visits. Clearly, the DFD model mitigates congestion and improves access rather than fully solving specialist shortages.

**Fig 1:**
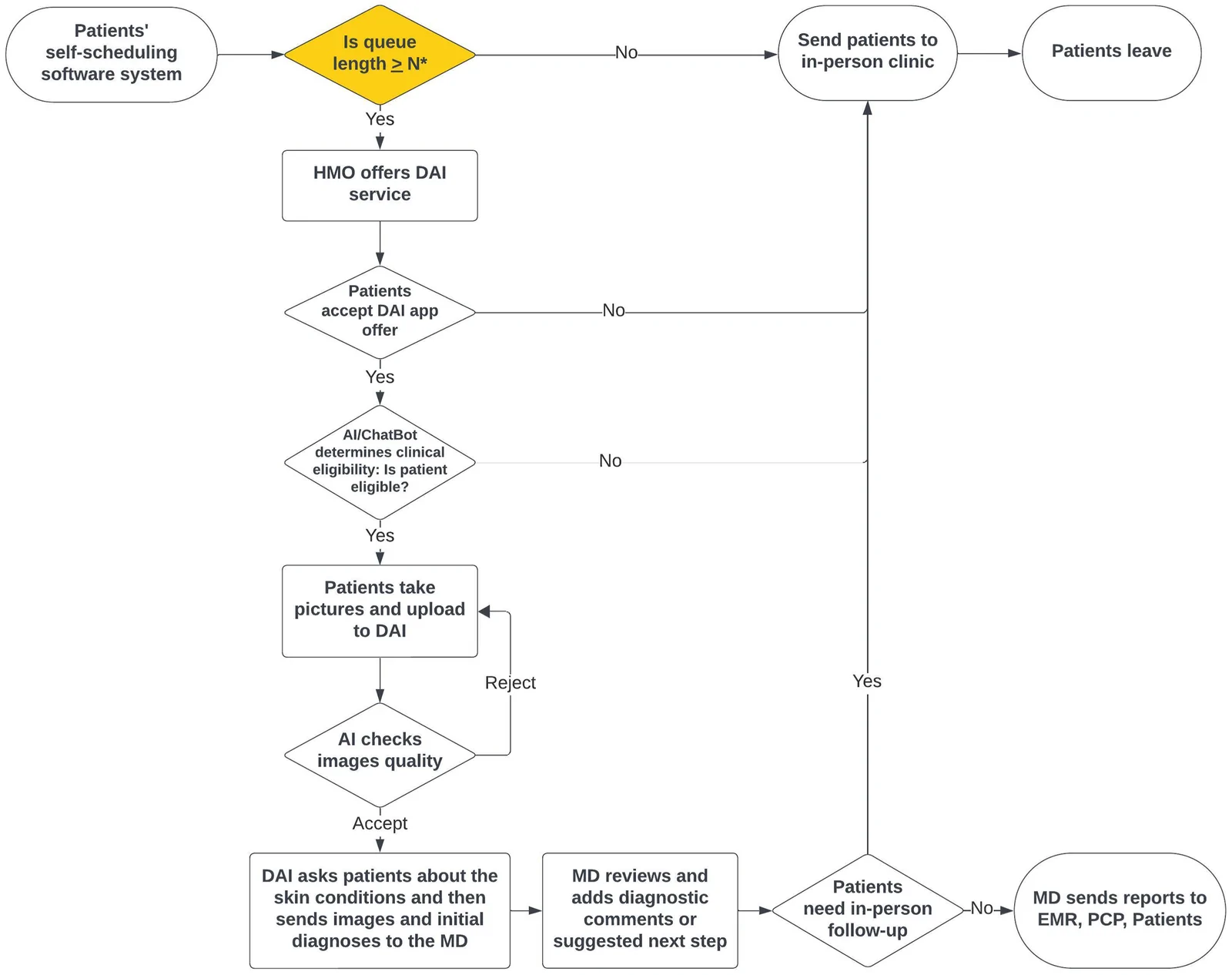
Case workflow for AI-assisted teledermatology surge capacity. Patients are offered the Dermatologic AI (DAI) pathway only when the in-person queue length reaches the diversion threshold *N*. The DAI app screens eligibility, collects clinical history, and guides image capture; then board-certified dermatologists review every case, and patients needing in-person evaluation are prioritized back into the clinic.

The central insight, supported by mathematical and operational analyses, is that an HMO dermatology clinic may need to divert only a small share of patients to reduce expected waiting times dramatically. Analyzing real data from a large HMO serving more than 2 million members, we observed that a typical urban in-person clinic received an average of 39 dermatology appointment requests per day and had the capacity to serve approximately 40 patients. This near-capacity schedule produced an observed average wait of about 20.4 working days, or 800 patients. Testing the proposed dynamic diversion approach on real-data calibrated simulations, we discovered that even when only 12% of patients use the remote pathway, the average in-person appointment queue will likely fall by more than 90%, as the simulated mean waiting time declines from about 20.5 to 1.6 working days; similar reductions have been reported.^4^ Meanwhile, the remote pathway should remain lightly loaded, with reports typically returned within 1 to 2 working days, consistent with the HMO’s service-level contract with the third-party medical group.

Our approach goes beyond simply adding teledermatology.^5^ We use advanced queueing and economic theories to identify the optimal queue threshold at which eligible patients are offered the option of opting to use AI-assisted teledermatology through an external provider group. This group serves as a reserve clinical capacity, absorbing demand surges without permanent HMO staffing or space expansion. Our simulation models also indicate that the DFD model will likely improve rural access to specialist care, given the uneven distribution of supply. Overall, it preserves in-person access while dramatically reducing waits. Still, future large-scale prospective studies must assess diagnostic accuracy, uptake, algorithmic bias, safety, equity, concordance, satisfaction, and costs.

## Conflicts of interest

None disclosed.
